# Examining the support needs of African and African‐Caribbean people living with dementia within Adult Social Care Services: an ethnographic study in the UK

**DOI:** 10.1002/alz70860_099792

**Published:** 2025-12-23

**Authors:** Shadreck Mwale, Andy Northcott, Juanita Hoe, Katie Featherstone

**Affiliations:** ^1^ Geller Institute of Ageing and Memory, the University of West London, London, London, United Kingdom; ^2^ Geller Institute of Ageing and Memory, University of West London, London, London, United Kingdom; ^3^ University of West London, London, United Kingdom

## Abstract

**Background:**

The UK population of ethnic minority people living with dementia is likely to increase sevenfold by 2050. For decades attempts to understand experiences of minority ethnic communities have typically grouped ‘minority ethnic communities’ together and have failed to recognise difference and diversity within and between communities. These approaches have further reinforced stereotypical narratives for understanding access to services, typically reporting ‘lack of knowledge’, cultural and religious beliefs, and language barriers among others, with findings typically placing the onus for change on marginalised communities themselves rather than services. Research addressing specific needs of African and African Caribbean people living with dementia is currently minimal, but suggests they are a population least likely to be recognised by services, experience historical discrimination, and significant health inequalities.

**Methods:**

This paper draws on ethnographic research informed by an intersectional approach. Intersectionality proffers making sense of and illustrating how forms of subjugation exist for marginalised vulnerable adults within institutional settings, therefore useful for analysing how class, gender and ethnicity intersect in people living with dementia, itself a stigmatised and silenced condition. The paper draws on data from discussion groups with a research steering group.

**Result:**

We will discuss findings from a recently established steering group of African and African Caribbean older people and people living with dementia to identify research priorities and outline the research co‐produced with them. This will examine the experiences of African and African Caribbean people living with dementia across a number of Local Authority sites in England. This in‐depth ethnographic study will follow the experiences of a group of African and African Caribbean people living with dementia and their care partners through a series of interviews and while also examining how social care staff recognize and respond to the needs of this population. This knowledge is vital to improving services, support the provision of culturally specific services, and to meet the needs of this population.

**Conclusion:**

We argue that rather than aggregating ethnic minority groups together, a focussed approach informed by intersectionality is required to better understand the diverse and complex needs of ethnic minority populations across the UK and Europe.

